# Independent Value of Cardiac Troponin T and Left Ventricular Global Longitudinal Strain in Predicting All-Cause Mortality among Stable Hemodialysis Patients with Preserved Left Ventricular Ejection Fraction

**DOI:** 10.1155/2014/217290

**Published:** 2014-05-07

**Authors:** Junne-Ming Sung, Chi-Ting Su, Yu-Tzu Chang, Yu-Ru Su, Wei-Chuan Tsai, Saprina P. H. Wang, Chun-Shin Yang, Liang-Miin Tsai, Jyh-Hong Chen, Yen-Wen Liu

**Affiliations:** ^1^Division of Nephrology and Cardiology, Department of Internal Medicine, College of Medicine and Hospital, National Cheng Kung University, 138 Sheng-Li Road, Tainan 704, Taiwan; ^2^Department of Human Genetics, Graduate School of Public Health, University of Pittsburgh, Pittsburgh, PA, USA; ^3^Public Health Sciences Division, Fred Hutchinson Cancer Research Center, Seattle, WA, USA; ^4^Division of Nephrology, Department of Internal Medicine, Catholic Fu-An Hospital, Yun-Lin, Taiwan

## Abstract

Using a speckle-tracking echocardiography (STE), we recently demonstrated that a left ventricular (LV) global longitudinal strain (GLS) ≥ −15% and the serum cardiac troponin T (cTnT) concentration are associated with mortality in stable hemodialysis patients with preserved LV ejection fraction (LVEF). In this study, we explored the relationship between cTnT and echocardiographic parameters and evaluated whether the prognostic value provided by cTnT is independent of a GLS ≥ −15% and vice versa. Eighty-eight stable hemodialysis patients with preserved LVEF were followed for 31 months. STE studies and measurements of cTnT were performed at baseline. CTnT concentration had a modest correlation with GLS (*r*_*s*_ = 0.44; *P* < 0.001) but had a weak or nonsignificant correlation with other echocardiographic parameters. Adjusting for clinical parameters, hazard ratios for each increase of 0.01 ng/mL in cTnT, and a GLS ≥ −15% on mortality were 1.13 (*P* = 0.009) and 3.09 (*P* = 0.03) without significant interaction between cTnT and GLS ≥ −15%. In addition, an increased cTnT concentration, a GLS ≥ −15%, or their combination showed significant additional predictive value for mortality when included in models consisting of clinical parameters. Therefore, both cTnT and a GLS ≥ −15% are independent predictors of mortality and are useful for risk stratification.

## 1. Introduction

Mortality in patients with end-stage renal diseases (ESRDs) remains high mainly because of their high cardiovascular disease burden [1–3]. The kidney disease outcome quality initiative (KDOQI) guidelines recommend that conventional echocardiography should be performed at the initiation of dialysis and every 3 years thereafter in all ESRD patients for cardiac risk stratification and optimization of therapies [4–7]. However, hemodialysis patients with heart failure (HF) and/or overt systolic dysfunction, defined by low left ventricular (LV) ejection fraction (LVEF) on conventional echocardiography, have very poor outcome [4–6, 8, 9] and frequently respond poorly to therapies [10]. It is thus feasible that early identification of high-risk patients in an asymptomatic and stable hemodialysis population with preserved LVEF may facilitate an early initiation of therapies to improve outcome.

For early detection of subclinical heart disease, two-dimensional speckle-tracking echocardiography (STE) with myocardial deformation (2D strain) analysis and the measuring of serum cardiac biomarkers, such as cardiac troponin T (cTnT), may be useful tools. STE with 2D strain analysis is a quantitative method for the assessment of subtle LV dysfunction, which cannot be evaluated by semiquantitative conventional echocardiography [11–16]. Using 2D strain analysis, LV global peak systolic longitudinal strain (GLS) or circumferential strain (CS) is the ratio of the maximal change in myocardial longitudinal or circumferential length in systole to the original length, respectively. During systole, the LV myocardium shortens in either direction; therefore, GLS or CS has a negative value, and less negative GLS or CS value indicates poorer global LV systolic function [13, 16]. GLS has been demonstrated to be a more sensitive predictor for all-cause mortality than LVEF in the general population [17]. We recently reported that a less negative GLS (defined as GLS ≥ −15%, i.e., an absolute value of GLS ≤ 15%) but not LVEF predicted all-cause and cardiac mortality among stable hemodialysis patients with preserved LVEF (LVEF ≥ 50%), indicating that GLS is a promising marker for early risk stratification [18]. As for serum cardiac biomarker, high circulating cTnT concentrations are associated with high mortality in dialysis patients [2, 19, 20]. The food and drug administration and the KDOQI guidelines [7] both indicate the use of cTnT as a biomarker for mortality risk stratification in dialysis patients. In our previous study, we also found that the elevated cTnT concentration correlated with GLS and is associated with high mortality in this hemodialysis population [18].

Validation of a novel marker for risk stratification in a specific population requires a phased approach. Early-phase studies should demonstrate that the novel marker is associated with the outcome. Midphase studies should explore the relationships between various markers and demonstrate that the new marker provides additional value beyond traditional and other markers in identifying high-risk patients and/or changing the decision-making process. The relationship between cTnT concentrations and conventional echocardiographic parameters has been extensively studied in a general dialysis population [2, 19–21]; however, the relationship between cTnT concentrations and subtle LV dysfunction and clinical characteristics in stable hemodialysis patients with preserved LVEF is still unclear, though we have noted that the cTnT concentration correlated with GLS in our previous study [18]. Furthermore, because cTnT might correlate with GLS, it raises a question of whether the association of cTnT with mortality remains significant after adjustment for other prognostic factors including GLS. In other words, whether cTnT can replace GLS in risk stratification, or vice versa, is still unknown. In addition, it remains unclear whether there is an additional prognostic value of cTnT or a GLS ≥ −15% or their combination beyond other prognostic factors. In this study, we explored the relationships between cTnT concentrations and patients' characteristics and STE-measured echocardiographic parameters and evaluated the additional prognostic value provided by cTnT or a GLS ≥ −15% or their interaction to define their clinical usefulness.

## 2. Subjects and Methods

### 2.1. Patients

This study adhered to the Declaration of Helsinki and all enrolled patients provided written informed consent. The study protocol was approved by the Human Research and Ethics Committee of our institute (IRB number: ER-98-073). As previously described [18], from December 2008 to January 2009, adult stable hemodialysis patients (≥18 years old) receiving a maintenance hemodialysis program consisting of 4 hours a session, thrice weekly for more than 3 months, were prospectively enrolled from two community hospitals in Yun-Lin County, Taiwan: National Cheng Kung University Hospital Dou-Liou Branch and Catholic Fu-An Hospital. In total, 120 hemodialysis patients without intercurrent or terminal illnesses were screened and 109 patients were included (3 with starting long-term hemodialysis <3 months and 8 without the willingness to participate). Additional patients were subsequently excluded because of old age (≥80 years, *n* = 3), LVEF <50% (*n* = 2), or episodic or persistent HF (≥NYHA FC III) [22, 23] within 6 months (*n* = 3). Patients with chronic atrial fibrillation (*n* = 4), recent infarction (*n* = 1), severe valvular heart disease (*n* = 2), or inadequate image quality (*n* = 6) were also excluded. The 88 enrolled patients were followed for 31 months or until death. Upon enrollment and during the follow-up period, clinical information on comorbidities, medical history, and cardiovascular medication were obtained by a careful review of each patient's medical record and/or a self-reported questionnaire. The primary outcome was all-cause mortality.

### 2.2. Biochemical Measurements

Blood was collected before the midweek dialysis session in the same week that the echocardiographic study was performed. Sera were stored at −80°C until analysis, when they were thawed for measurement of the concentrations of cTnT (4th generation Troponin T STAT immunoassay, ElecSys 2010 System, Roche Diagnostics, Indianapolis, IN, USA), high-sensitivity C-reactive protein (BN II analyzer; Dade Behring, Glasgow, DE, USA), interleukin-6 (chemiluminescent sandwich ELISA, Quantikine Human interleukin-6; R&D Systems Inc., Minneapolis, MN, USA), and procollagen type I C-terminal peptide (Takara Bio Inc., Otsu, Shiga, Japan). The measurements of cTnT concentration were performed in the clinical laboratory at National Cheng-Kung University Hospital and were supervised by one of the coauthors (YWL). CTnT concentrations were assayed in batch using the automated ElecSys 2010 analyzer. The lower limit of detection for this assay is 0.01 ng/mL, which also reflects the 99th percentile cutoff limit for detection of myocardial necrosis. In our laboratory, the coefficient of variation (CV) at 0.078 ng/mL is 5.0% and the CV at 2.300 ng/mL is 2.5%. Serum cholesterol, triglycerides, calcium, phosphate, and albumin were measured using an automatic analyzer.

### 2.3. Echocardiographic Measurements

All patients were examined by one well-trained cardiologist (YWL, with 10 years of experience in echocardiographic examination) using an ultrasound system with a 3.5 MHz probe (Vivid-i, GE Healthcare, Horten, Norway). Two-dimensional STE and tissue Doppler imaging (TDI) were performed as previously described [15, 16, 18]. All participants received an echocardiographic examination at the halfway point of the hemodialysis session (the second or third hour of each session) [18]. We measured LVMi, LV volume, LVEF, and left atrial volume index, and LV hypertrophy (LVH) was defined as an LVMi >115 g/m^2^ for men and >95 g/m^2^ for women [22–24].

Using pulsed-wave Doppler, we measured the peak early (E)-wave and late (A)-wave velocities of the mitral inflow. The pulse TDI of the mitral annulus movement was acquired from the apical 4-chamber view when a sample volume was placed first at the septal side and then at the lateral side of the mitral annulus. To obtain the peak systolic (*s*′) and early diastolic (*e*′) velocities, we measured 3 end-expiratory beats and averaged these values for further analysis. We used the average *e*′ velocity acquired from the septal and lateral sides of the mitral annulus to calculate the ratio of the mitral inflow *E* to the *e*′ velocity (average *E*/*e*′ = *E*/[(*e*_septal_′ + *e*_lateral_′)/2]). We acquired 2D gray-scale STE images in the 3 standard apical views (i.e., apical 4-chamber, apical 2-chamber, and apical long-axis) for 3 cardiac cycles and then stored the images digitally for subsequent off-line analysis. To evaluate the fluid status, we measured the IVC diameter twice with an average value defined as the IVCe at the end of expiration in a subxiphoid location and just proximal to the junction of the hepatic veins [16, 22, 23, 25]. IVCe > 1.53 cm indicates hypervolemia in ESRD patients [16, 25].

### 2.4. 2D Strain Analysis

Off-line 2D strain analysis was performed using automated functional imaging software (EchoPAC work station, BT09, GE Healthcare, Israel). Peak systolic longitudinal strain was automatically obtained from the 3 standard apical views. The average peak systolic longitudinal strain value from the 3 apical views was regarded as the GLS. Six LV segments on the parasternal short-axis view at the midpapillary level were examined to obtain the segmental circumferential strains in systole and the average of these 6 segmental circumferential strain was defined as the CS [16, 18]. The Bland-Altman analysis revealed no systemic bias of the GLS between intra- and interobserver agreements [18]. In addition, hemodialysis per se did not affect GLS measurement [18].

### 2.5. Statistical Analysis

Continuous data are presented as the mean ± standard deviation, or the median and interquartile range, depending on the distribution. Dichotomous data are presented as numbers and percentages. We stratified patients by tertiles of the cTnT concentration and comparisons among groups across the tertiles were performed using the trend test. A Kruskal-Wallis test was used for nonnormally distributed data. The Kaplan-Meier method with a log-rank test was used to compare mortality between strata. The relationships among continuous variables were evaluated using a Pearson correlation or a Spearman's correlation analysis depending on the distribution. Uni- and multivariate Cox regression analysis were used to examine the risk factors for all-cause mortality. We confirmed that all variables considered in the regression analysis met the assumption of proportional hazards. The additional predictive value of cTnT concentrations and a GLS ≥ −15% for mortality was investigated using a multivariate Cox regression with analysis of the deviance between different models and a receiver operating characteristic (ROC) curve analysis. A *P* < 0.05 was considered statistically significant. All statistical analyses were performed using SAS software, version 9.2 (SAS Institute) or SPSS (Statistical Package for the Social Sciences) software, version 17.0 (SPSS Inc.).

## 3. Results

This was a prospective study of 88 stable hemodialysis patients with preserved LVEF (LVEF ≥ 50%). All patients presented with anuria and received adequate hemodialysis (average Kt/V, 1.71 ± 0.23; hemoglobin, 10.2 ± 1.2 mg/dL) [26]. The baseline characteristics and echocardiographic parameters in the all enrolled patients had been reported in a previous study [18]. Patients were stratified by tertiles based on their cTnT concentrations: lower (cTnT ≤ 0.02 ng/mL), middle (0.02 < cTnT ≤ 0.042 ng/mL), and upper tertiles (cTnT > 0.042 ng/mL). Comparisons of baseline characteristics across tertiles of cTnT concentrations are listed in Table 1. The prevalence of diabetes was significantly different across tertiles but not background CAD, hypertension, and LVH. Other variables were not significantly different across tertiles. Comparisons of baseline LV geometric and functional parameters across tertiles of cTnT concentrations were listed in Table 2. Only LV-GLS showed a significant difference across tertiles; other variables, including LV mass index (LVMi), LV diastolic functional parameters, and other LV systolic parameters, did not. There was no significant difference in LV end-diastolic volume index (LVEDVi) or end-expiratory inferior vena cava diameter (IVCe) across tertiles, and the IVCe did not increase in the patients of each group, indicating that the volume status of patients in these three groups was similar and that substantial hypervolemia did not occur [16, 27]. In total, 82 patients (93%) presented LVH [22], and all three groups presented an inverse ratio between early and late LV filling velocity, a high *E*/*e*′, and a high left atrial volume index (LAVi), indicating that most patients in this cohort had LVH and diastolic dysfunction.

There were different degrees of correlations among the echocardiogram parameters LVMi, LVEF, GLS, systolic longitudinal strain rates (LSRs), CS, and systolic circumferential strain rates (CSRs) (see Table S1 in Supplementary Material available online at http://dx.doi.org/10.1155/2014/217290). Because the distribution of cTnT was skewed (skewness = 0.98), we assessed the relationships between cTnT and echocardiographic parameters using Spearman's correlation (Table 3). CTnT concentrations modestly correlated with GLS (*r*_*s*_ = 0.44; *P* < 0.001) and weakly correlated with LVEF, LSRs, CS, and CSRs (all |*r*_*s*_| < 0.3 and all *P* < 0.05). Other variables, including LVMi, LVEDVi, IVCe, and diastolic functional indicators had no significant correlation with cTnT concentrations.

**Figure 1:**
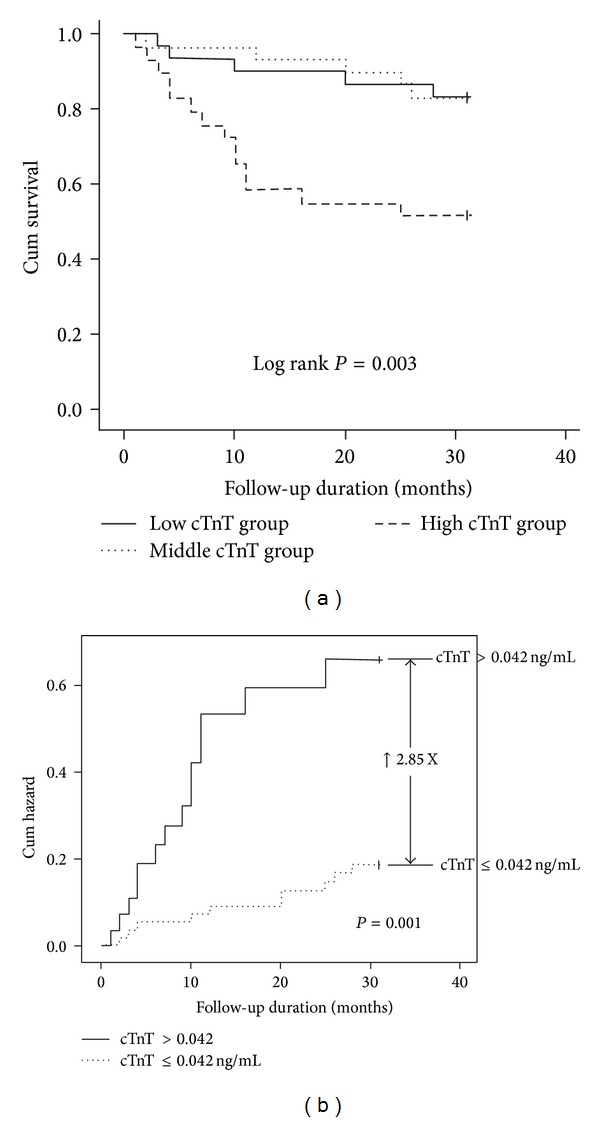
(a) The Kaplan-Meier estimates of the overall survival probability of patients stratified by tertiles of cTnT concentrations. (b) The Kaplan-Meier estimates of the cumulative hazard rate for all-cause mortality stratified by the cTnT concentration with a cutoff of 0.042 ng/dL (the lower limit of the upper tertile) with a hazard ratio (HR) of 2.85 (95% confidence interval [CI], 1.44–5.62; *P* = 0.001) for cTnT > 0.042.

During the follow-up of 31 months, 24 patients (27.3%) died: 9 from cardiovascular death, 11 from infections, and 4 from liver disease [18]. The baseline median plasma cTnT concentrations were significantly higher in patients who died than in those who survived to the end of 31-month follow-up (0.049 [25th, 75th percentiles: 0.023, 0.134]* versus* 0.025 [0.010, 0.042], *P* = 0.001).  Figure 1(a) illustrates the Kaplan-Meier estimates of the overall survival probability of patients stratified by tertiles of cTnT concentrations.  Figure 1(b) shows the Kaplan-Meier estimates of the cumulative hazard rate for mortality stratified by the cTnT concentration with a cutoff of 0.042 (the lower limit of the upper tertile) with a hazard ratio (HR) of 2.85 (95% confidence interval [CI], 1.44–5.62; *P* = 0.001) for cTnT > 0.042. The results of the univariate Cox regression analysis for mortality are listed in Table S2. Based on our previous study [18] and a recent meta-analysis [28], we used a GLS of −15% and a CS of −23.3% as cutoff values in this analysis. In addition, because the plasma values of cTnT were low and an increase of 0.01 in the cTnT concentration may be clinically significant, we multiplied the cTnT values by 100 (cTnT × 100) for analysis. For the multivariate Cox regression analysis, a background of CAD, diabetes, and hypertension together with plasma albumin concentration, which were significant predictors in the univariate analysis, were selected to be basic clinical parameters and to construct the basic model (Table 4). Because the cTnT concentrations were modestly correlated with GLS, we first observed the changes in HR when adding cTnT × 100 and GLS ≥ −15% individually or concomitantly in the basic model. We observed no substantial change in HR of cTnT × 100 and GLS ≥ −15%. Furthermore, there was no significant interaction between cTnT × 100 and a GLS ≥ −15% in the full model (Table 4), indicating that cTnT and a GLS ≥ −15% are independently associated with mortality. After adjustment for basic clinical parameters, the HRs associated with an increase of 0.01 in cTnT concentration and a GLS ≥ −15% in relation to mortality were 1.13 (1.03–1.24; *P* = 0.009) and 3.09 (1.14–8.43; *P* = 0.03), respectively (Table 4, full model). We subsequently performed a multivariate Cox regression analysis with backward elimination. In the final model (Table 4, reduced model 2), cTnT × 100, a GLS ≥ −15%, and serum albumin were significant predictors of mortality, whereas background hypertension was a marginally significant predictor. There was no significant interaction between cTnT × 100 and a GLS ≥ −15% in the final model. The 95% CIs of each HR in the Cox regression models shown in Table 4 are listed in Table S3.

The additional predictive value of cTnT and GLS for all-cause mortality was investigated using a multivariate Cox regression with an analysis of deviance between different models and an ROC curve analysis (Table 5). Using these two analysis methods, mortality was best predicted when the cTnT concentration and a GLS ≥ −15% were simultaneously included in the model incorporating the basic clinical parameters. In addition, including either the cTnT concentration or a GLS ≥ −15% in the model incorporating the basic clinical parameters also increased the predictive power of the model.

## 4. Discussion

The principal findings of the current study were that both the cTnT concentration and a GLS ≥ −15% were independent and significant predictors of all-cause mortality, despite the modest correlation between cTnT and GLS, and added incremental predictive value in determining the risk of mortality beyond basic clinical parameters (background CAD, diabetes and hypertension, and serum albumin concentrations) in stable hemodialysis patients with preserved LVEF. The prognostic predictive value of the cTnT concentration could not be replaced by GLS and vice versa.

### 4.1. Relationship between cTnT Concentration and Baseline Echocardiographic Parameters and Clinical Characteristics

Despite contradictory findings [2], elevated cTnT concentrations have been linked to LVH, LV dilation, and systolic and diastolic dysfunction in hemodialysis patients without cardiovascular symptoms [20, 21, 29–31]. In the present study, the cTnT concentration was not significantly correlated with diastolic functional parameters or LVMi, possibly because the diastolic dysfunction and LVH were present in the majority of the enrolled patients. This finding of lacking a significant association between the cTnT concentration and LVMi is similar to the study by DeFilippi et al. [2] that also had high prevalence of LVH in their enrolled patients. The most noteworthy finding was that subtle LV systolic dysfunction, especially represented as the GLS, was modestly correlated with cTnT concentrations. The cTnT concentration has also a significant but weak association with other systolic functional parameters including LVEF, LSRs, CS, and CSRs (Table 3). This finding indicates that the elevated cTnT concentration may, at least in part, reflect subtle LV dysfunction but not LV diastolic dysfunction or mass in this patient population with high prevalence of LVH and diastolic dysfunction. One possible explanation for this result is that some patients with subtle LV dysfunction, detected by measuring the GLS but not other systolic parameters, have concomitant subclinical myocyte injury and/or stress resulting in the increase of cTnT. Furthermore, myocardial stunning may contribute to the subsequent development of systolic dysfunction in fixed segment(s) even without reduction of LVEF in hemodialysis patients [32, 33], resulting in a less negative GLS. In addition, it has been demonstrated that a high cTnT concentration is independently associated with the presence or new development of myocardial stunning [34]. This information indicates that elevated cTnT concentration and less negative GLS possibly share some common underlying mechanisms.

There was no significant difference in the prevalence of CAD across tertiles of cTnT concentrations in the current study. Some studies [2, 35, 36] have suggested an association between cTnT and CAD but others have failed to demonstrate any correlation [29, 30, 37], implying that CAD is not the sole causal factor for the increased cTnT concentrations. However, we did find that dialysis patients with diabetes had significantly elevated serum cTnT concentrations. Several studies have indicated that diabetes is associated with raised cTnT concentrations because of the presence of cardiac microvascular disease [29, 38–40]; in contrast, Ooi and House [41] suggested that this association may result from protein glycosylation, alteration of the degradation of the molecules, and/or reexpression of fetal genes.

### 4.2. Association of Outcomes and Prognostic Predictive Values

Elevated cTnT concentration is a powerful prognostic predictor in the ESRD population [19, 42–47]. Several studies have reported associations between elevated cTnT concentration and LVMi and LV dysfunction [2, 19, 20, 31, 48]; however, elevated cTnT concentration remains associated with mortality after adjustment for LVMi in hemodialysis patients [20] and after adjustment for LVMi and LVEF in peritoneal dialysis patients [19], indicating that the prognostic value of cTnT reflects more than just LV mass and function. In the present study, LVH and LVMi were not significant predictors possibly because most of the enrolled patients had LVH. It should be noted that most stable hemodialysis patients have LVH and preserved LVEF [2, 29], and our finding indicates that cTnT concentrations and GLS are useful indicators in evaluating the prognosis in such a population.

We found that cTnT concentrations and a GLS ≥ −15%, but not LVEF or other systolic echocardiogram parameters, were independently associated with mortality. Although GLS modestly correlated with LVEF (Table S1), the LVEF reflects LV radial and transverse function and only partially reflects longitudinal function, whereas GLS mainly reflects LV longitudinal function [17, 49]. LV longitudinal function is largely determined by the subendocardial region, and GLS may thus be more sensitive in detecting the presence of pathological conditions such as myocardial ischemia or fibrosis [17, 49, 50], which might explain the predictive value of GLS for long-term prognosis in hemodialysis patients with preserved LVEF. The reasons for the differences in outcome prediction of cTnT and less negative GLS are unclear. CTnT is a cardiac injury marker, whereas GLS is a functional marker, and our findings suggest they may capture different or residual risks associated with poor outcomes.

The prognostic value of elevated cTnT concentrations and GLS ≥ −15% might not be attributed to background diabetes or CAD as demonstrated by the finding that no substantial change in the HRs of cTnT and a GLS ≥ −15% was found when cTnT and a less negative GLS were added to the model, regardless of whether the model was adjusted for background CAD and diabetes (full model and reduced models 1 and 2 in Table 4).

### 4.3. Possible Clinical Implications

Given that cTnT concentrations and a GLS ≥ −15% have additional prognostic value beyond conventional echocardiographic and clinical parameters, echocardiography (with STE) should be performed in conjunction with measurement of cTnT concentrations for early risk stratification in stable hemodialysis patients with preserved LVEF.

An STE study includes longitudinal, radial, and circumferential strains and twist measurements; the thorough strain measurement is complicated and may be time-consuming, especially for inexperienced operators. All STE-measured parameters can be collected in research studies; however, the measuring GLS is particularly useful because it appears to be highly sensitive, relevant for prognosis, and more reproducible than either circumferential or radial strain in the general population [51, 52] and in stable hemodialysis patients with preserved LVEF. To simplify the strain examination in clinical practice, assessment of only the longitudinal strain may be used to facilitate the analysis. With this kind of modified STE analysis, the measurement of GLS generally only requires 2–4 minutes and may be feasible as a component of routine echocardiographic examinations in clinical practice [53].

### 4.4. Study Limitations

The current study has several limitations. First, the number of enrolled patients was limited; therefore this study may not have had sufficient power to explore all of the factors associated with mortality. In addition, we were not able to analyze the impact of cTnT concentrations and GLS ≥ −15% on cause-specific mortality, such as cardiovascular mortality. With the current sample size, however, we already had sufficient statistical power to detect significant effects of cTnT concentrations and a GLS ≥ −15% on all-cause mortality, and, therefore, the findings were statistically significant. Nevertheless, a study with a larger sample size will enable the simultaneous evaluation of more predictors in the future and may help confirm our findings. Second, patients with low LVEF (<50%) and/or HF were not enrolled in the current study, which limits the generalizability of our findings to a general hemodialysis population. However, the serum cTnT concentration [54–56] and GLS [57–59] were predictive of the prognosis in nondialysis acute and chronic HF patients with varying LVEF values. Whether the measurement of cTnT concentrations or GLS or the combination thereof is also useful for risk stratification in a general hemodialysis population including patients with low LVEF and/or HF warrants further studies. Third, some dialysis patients had severe valvular heart diseases, atrial fibrillation, or poor echocardiographic image quality, which excluded the possibility of GLS analysis. Finally, we did not measure brain natriuretic peptide concentrations in this study.

### 4.5. Conclusions

Both the cTnT concentration and a GLS ≥ −15% are powerful, independent predictors of all-cause mortality and add prognostic value to the clinical and conventional echocardiographic parameters in current use thus enabling the early identification of high-risk patients and inform clinical decision making among stable hemodialysis patients with preserved LVEF. Further studies are warranted to define effective and early intervention strategies for these high-risk patients.

## Supplementary Material

Supplemental Table S1: Pearson correlations among Echocardiographic parameters.
Supplemental Table S2: Univariate analysis of factors related to all-cause mortality.

## Figures and Tables

**Table 1:** Baseline clinical characteristics of stable hemodialysis patients with preserved left ventricular ejection fraction.

	cTnT Tertiles			*P* for trend
	Lower (cTnT ≤ 0.02 ng/mL, *n* = 30)	Middle (0.02 < cTnT ≤ 0.042 ng/mL, *n* = 29)	Upper (cTnT > 0.042 ng/mL, *n* = 29)	
Age (years)	64.0 ± 13.0	67.9 ± 8.7	68.4 ± 11.6	0.27
Male, *n* (%)	11 (37%)	9 (31%)	12 (42%)	0.71
BMI (kg/m^2^)	21.3 ± 3.0	22.6 ± 2.5	21.2 ± 2.9	0.15
Kt/v	1.75 ± 0.22	1.71 ± 0.24	1.66 ± 0.22	0.30
IDWG, (kg)	2.63 ± 0.83	2.92 ± 1.02	2.78 ± 1.25	0.60
IDWG, (%)	5.1 ± 1.8	5.2 ± 1.6	5.1 ± 2.3	0.98
Hemodialysis duration (years)	6.5 (4, 9)	4.4 (2, 9.9)	4 (1.8, 6.4)	0.31
SBP (mmHg)	143.0 ± 13.2	152.2 ± 16.3	144.1 ± 15.2	0.93
DBP (mmHg)	73.9 ± 8.7	81.0 ± 7.8	76.2 ± 10.2	0.44
Heart rate	74.7 ± 11.4	75.8 ± 12.4	75.5 ± 12.6	0.94
Prevalent CAD	8 (27%)	11 (38%)	12 (41%)	0.46
Diabetes mellitus	7 (23%)	16 (55%)	22 (76%)	<0.001*
Hypertension	27 (90%)	25 (86%)	25 (86%)	0.85
LV hypertrophy^#^	28 (93%)	27 (93%)	27 (93%)	0.95
ACEI/ARB	17 (57%)	18 (62%)	13 (45%)	0.40
*β*-Blocker	15 (50%)	11 (38%)	15 (52%)	0.40
CCB	15 (50%)	17 (59%)	18 (62%)	0.48
Statin	6 (20%)	4 (14%)	5 (17%)	0.94
Calcium (mg/dL)	9.4 ± 0.9	9.1 ± 0.7	9.2 ± 0.7	0.26
Phosphate (mg/dL)	4.4 ± 1.3	4.8 ± 1.3	4.1 ± 1.1	0.19
Albumin (g/dL)	3.3 ± 0.5	3.4 ± 0.3	3.2 ± 0.4	0.21
Cholesterol (mg/dL)	163.1 ± 35.7	168.6 ± 41.5	157.6 ± 35.7	0.55
hsCRP (mg/dL)	0.26 (0.14, 0.63)	0.66 (0.18, 0.99)	0.45 (0.19, 1.95)	0.12
IL-6 (pg/mL)	9.5 (7.3, 16.3)	9.7 (6.9, 13.7)	10.6 (6.3, 19.1)	0.85
PICP (ng/mL)	843.6 ± 398.5	802.1 ± 331.8	952.8 ± 444.9	0.34

Continuous data are expressed as the mean ± standard deviation or the median (25th and 75th percentiles); categorical data are expressed as the number (percentage). A nonparametric Kruskal-Wallis test was used for nonnormally distributed data.

*P < 0.05; ^#^LV hypertrophy was diagnosed by echocardiography.

Abbreviations: ACEI: angiotensin-converting enzyme inhibitor; ARB: angiotensin II-receptor blocker; BMI: body mass index; CAD: coronary artery disease; CCB: calcium channel blocker; cTnT: cardiac troponin T; DBP: diastolic blood pressure; hsCRP: high-sensitivity C-reactive protein; IDWG: interdialytic weight gain; IL: interleukin; Kt/V: an indicator of dialysis adequacy (*K*: urea clearance; *t*: dialysis time; *V*: urea distribution volume); LV: left ventricle; PICP: procollagen type I C-terminal peptide; SBP: systolic blood pressure.

**Table 2:** Baseline echocardiographic study of asymptomatic hemodialysis patients with preserved left ventricular ejection fraction.

	cTnT Tertile			*P* for trend
	Lower (cTnT ≤ 0.02 ng/mL, *n* = 30)	Middle (cTnT of 0.02–0.042 ng/mL, *n* = 29)	Upper (cTnT > 0.042 ng/mL, *n* = 29)	
LV EDVi (mL/m^2^)	69.9 ± 18.8	69.5 ± 20.8	71.1 ± 19.3	0.96
LVMi (gm/m^2^)	135.4 ± 25.0	151.5 ± 57.2	159.1 ± 64.7	0.27
IVCe diameter (cm)	1.21 ± 0.26	1.3 ± 0.21	1.35 ± 0.35	0.18
LVEF (%)	65.7 ± 5.2	64.7 ± 5.9	62.3 ± 6.4	0.10
*s*′ (cm/sec)	8.7 ± 2.0	8.8 ± 1.6	7.9 ± 2.2	0.16
GLS (%)	−20.0 ± 3.5	−17.6 ± 3.0	−16.4 ± 4.6	0.002*
LSRs (sec^−1^)	−1.02 ± 0.21	−0.98 ± 0.22	−0.89 ± 0.21	0.06
CS (%)	−22.2 ± 5.6	−20.7 ± 6.3	−19.6 ± 5.9	0.33
CSRs (sec^−1^)	−2.05 ± 0.56	−1.98 ± 0.66	−1.66 ± 0.45	0.05
*E* (m/sec)	0.79 ± 0.31	0.81 ± 0.31	0.79 ± 0.29	0.97
*A* (m/sec)	1.01 ± 0.28	1.09 ± 0.39	1.00 ± 0.27	0.53
*E*/*A*	0.85 ± 0.53	0.80 ± 0.35	0.78 ± 0.23	0.78
*e*′ (cm/sec)	5.0 ± 1.4	4.8 ± 1.1	4.7 ± 1.5	0.63
*E*/*e*′	15.8 ± 5.9	18.2 ± 10.1	16.8 ± 6.1	0.52
LAVi (mL/m^2^)	34.1 ± 7.9	35.6 ± 7.7	36.4 ± 8.9	0.67

Continuous data are expressed as the mean ± standard deviation or the median (25th and 75th percentiles); categorical data are expressed as the number (percentage). A nonparametric Kruskal-Wallis test was performed for nonnormally distributed data.

*P < 0.05.

Abbreviations: CS: average circumferential strain; CSRs: circumferential systolic strain rate; cTnT: cardiac troponin T; EDVi: end-diastolic volume index; EF: ejection fraction; *E*/*e*′: early transmitral velocity to tissue Doppler mitral annular early diastolic velocity ratio; GLS: global left ventricular peak systolic longitudinal strain; IVCe: end-expiratory inferior vena cava diameter; LAVi: left atrial volume index; LSRs: longitudinal systolic strain rate; LV: left ventricular; LVMi: left ventricular mass index; *s*′: left ventricular systolic myocardial velocity.

**Table 3:** Spearman's correlation between cardiac troponin T (cTnT) concentrations and echocardiographic parameters.

Variables	*r*_*s*_	*P*	Variables	*r*_*s*_	*P*
LVEDVi	0.10	0.44	IVCe (cm)	0.209	0.07
LVMi	0.18	0.14			

Systolic function			Diastolic function		
LVEF (%)	−0.23	0.04*	Average *E*/*e*′	0.05	0.66
GLS (%)	0.44	<0.001*	*e*′ (cm/s)	−0.14	0.20
LSRs (sec^−1^)	0.28	0.01*	*E* (m/s)	−0.08	0.46
CS (%)	0.23	0.049*	*A* (m/s)	−0.09	0.44
CSRs (sec^−1^)	0.28	0.02*	*E*/*A*	−0.01	0.95
*s*′ (cm/s)	−0.10	0.39	LAVi	0.14	0.31

*P < 0.05.

Abbreviations: CS: average circumferential strain; CSRs: circumferential systolic strain rate; cTnT: cardiac troponin T; EDVi: end-diastolic volume index; EF: ejection fraction; *E*/*e*′: early transmitral velocity to tissue Doppler mitral annular early diastolic velocity ratio; GLS: global left ventricular peak systolic longitudinal strain; IVCe: end-expiratory inferior vena cava diameter; LAVi: left atrial volume index; LSRs: longitudinal systolic strain rate; LV: left ventricular; LVMi: left ventricular mass index; SRs: systolic strain rate; *s*′: left ventricular systolic myocardial velocity.

**Table 4:** Cox regression analysis for all-cause mortality.

	Basic model		Basic model + cTnT × 100		Basic model + GLS ≥ −15%		Full model		Full model + interaction term		Reduced model 1		Reduced model 2 (final model)		Final model + interaction term	
	HR	*P*	HR	*P*	HR	*P*	HR	*P*	HR	*P*	HR	*P*	HR	*P*	HR	*P*
Albumin	0.17	0.004*	0.22	0.10	0.16	0.003*	0.23	0.047*	0.28	0.09	0.22	0.04*	0.24	0.048*	0.29	0.09
CAD	2.62	0.04*	2.43	0.04*	1.96	0.16	2.12	0.12	2.22	0.11	2.21	0.10	—	—	—	—
DM	3.17	0.03*	2.10	0.35	2.97	0.04*	1.72	0.40	1.49	0.50	—	—	—	—	—	—
HTN	3.39	0.02*	2.94	0.05*	3.09	0.02*	3.09	0.03*	2.64	0.09	3.00	0.04*	2.70	0.06	2.31	0.12
cTnT × 100	—	—	1.16	0.001*	—	—	1.13	0.009*	1.19	0.01*	1.15	0.001*	1.14	0.002*	1.21	0.001*
GLS ≥ −15%	—	—	—	—	3.57	0.02*	3.09	0.03*	4.00	0.07	3.37	0.02*	2.79	0.049*	6.45	0.01*
(cTnT × 100) : (GLS ≥ −15%)^#^	—	—	—	—	—	—	—	—	0.91	0.22	—	—	—	—	0.90	0.14

Data presented are based on the Cox regression analysis; 95% CIs for each HR are presented in Supplementary Table S3. In multivariate analysis, we firstly constructed the model including background coronary arterial disease (CAD) and diabetes and hypertension together with serum albumin concentration as a basic model; then, we added cTnT × 100 and/or a GLS ≥ −15% into the basic model to study their effects on mortality. Next, a backward stepwise procedure was used to choose the final reduced model, with variables significant at *P* < 0.10 being retained in the model. To obtain an adequate reduced model, before dropping a covariate from the model, we confirmed that its absence did not result in a substantial change in the overall predicting power of the model.

*P < 0.05;^ #^(cTnT × 100) : (GLS ≥−15%), interaction between cTnT × 100 and GLS ≥ −15%.

Abbreviations: CAD: coronary arterial disease; cTnT: cardiac troponin T, DM: diabetes; GLS: global left ventricular peak systolic longitudinal strain (a less negative GLS was defined by a GLS ≥−15%).

**Table 5:** Additional predictive values of cTnT × 100 and less negative GLS (≥−15%) for mortality using Cox regression models analysis and ROC curve analysis.

The additional value of cTnT × 100 and less negative GLS (≥−15%) in model prediction for mortality using Cox regression models analysis with analysis of deviance			The additional predictive value of cTnT × 100 and less negative GLS (≥−15%) based on ROC curve analysis with calculated AUCs				

	Chi-square	*P*		AUC	95% CI	*P* ^#^	*P* ^##^

Albumin + hypertension + GLS ≥ −15% versus albumin + hypertension + GLS ≥−15% + cTnT × 100	9.88	0.002*	Albumin + hypertension	0.68	(0.53–0.81)	—	—
Albumin + hypertension + cTnT × 100 versus albumin + hypertension + cTnT × 100 + GLS ≥ −15%	4.56	0.05*	Albumin + hypertension + GLS ≥ −15%	0.77	(0.65–0.88)	0.03*	—
			Albumin + hypertension + cTnT × 100	0.78	(0.6–0.89)	0.02*	—
			Albumin + hypertension + GLS ≥ −15% + cTnT × 100	0.85	(0.75–0.94)	<0.001*	—

Basic model versus basic model + GLS ≥ −15%	4.51	0.03*	Basic model	0.74	(0.62–0.86)	—	—
Basic model versus basic model + cTnT × 100	9.87	0.003*	Basic model + GLS ≥ −15%	0.81	(0.69–0.91)	—	0.04*
Basic model + GLS ≥ −15% versus basic model + GLS ≥ −15% + cTnT × 100	6.90	0.009*	Basic model + cTnT × 100	0.82	(0.71–0.91)	—	0.03*
Basic model + cTnT × 100 versus basic model + cTnT × 100 + GLS ≥ −15%	4.53	0.046*	Basic model + GLS ≥ −15% + cTnT × 100	0.89	(0.77–0.95)	—	0.001*
Basic model versus basic model + GLS ≥ −15% + cTnT × 100	11.40	0.002*					

First, we included background hypertension and plasma albumin concentration but excluded diabetes and coronary arterial disease based on final reduced model of Table 4 and studied the additional predictive values of cTnT × 100 and less negative GLS (≥−15%) to mortality. Then, we included diabetes and coronary arterial disease in addition to hypertension and serum albumin concentration to construct the basic model because they are commonly used for risk stratification in the hemodialysis population, and studied the additional predictive values of cTnT × 100 and less negative GLS (≥−15%) to mortality. *P < 0.05; ^ #^comparing the AUC of indicated model with that of the model including background hypertension and plasma albumin concentration; ^##^comparing the AUC of indicated model with that of the model including background coronary arterial disease, diabetes and hypertension, and plasma albumin concentration.

Abbreviations: AUC: area under curve; CI: confidence interval; cTnT: cardiac troponin T; GLS: global left ventricular peak systolic longitudinal strain.

## References

[1] Wen CP, Cheng TYD, Tsai MK (2008). All-cause mortality attributable to chronic kidney disease: a prospective cohort study based on 462 293 adults in Taiwan. *The Lancet*.

[2] DeFilippi C, Wasserman S, Rosanio S (2003). Cardiac troponin T and C-reactive protein for predicting prognosis, coronary atherosclerosis, and cardiomyopathy in patients undergoing long-term hemodialysis. *Journal of the American Medical Association*.

[3] Yang W-C, Hwang S-J (2008). Incidence, prevalence and mortality trends of dialysis end-stage renal disease in Taiwan from 1990 to 2001: the impact of national health insurance. *Nephrology Dialysis Transplantation*.

[4] Parfrey PS, Foley RN, Harnett JD, Kent GM, Murray DC, Barre PE (1996). Outcome and risk factors for left ventricular disorders in chronic uraemia. *Nephrology Dialysis Transplantation*.

[5] Zoccali C, Benedetto FA, Mallamaci F (2004). Prognostic value of echocardiographic indicators of left ventricular systolic function in asymptomatic dialysis patients. *Journal of the American Society of Nephrology*.

[6] Wang AY-M, Wang M, Lam CW-K, Chan IH-S, Lui S-F, Sanderson JE (2011). Heart failure in long-term peritoneal dialysis patients: a 4-Year prospective analysis. *Clinical Journal of the American Society of Nephrology*.

[7] (2005). K/DOQI clinical practice guidelines for cardiovascular disease in dialysis patients. *American Journal of Kidney*.

[8] Harnett JD, Foley RN, Kent GM, Barre PE, Murray D, Parfrey PS (1995). Congestive heart failure in dialysis patients: prevalence, incidence, prognosis and risk factors. *Kidney International*.

[9] Soucie JM, McClellan WM (1996). Early death in dialysis patients: risk factors and impact on incidence and mortality rates. *Journal of the American Society of Nephrology*.

[10] Wang AY-M, Sanderson JE (2011). Treatment of heart failure in long-term dialysis patients: a reappraisal. *American Journal of Kidney Diseases*.

[11] Zoccali C, Benedetto FA, Tripepi G (2006). Left ventricular systolic function monitoring in asymptomatic dialysis patients: a prospective cohort study. *Journal of the American Society of Nephrology*.

[12] Wang AY, Wang M, Lam CW, Chan IH, Lui SF, Sanderson JE (2013). Heart failure with preserved or reduced ejection fraction in patients treated with peritoneal dialysis. *American Journal of Kidney Diseases*.

[13] Gorcsan J, Tanaka H (2011). Echocardiographic assessment of myocardial strain. *Journal of the American College of Cardiology*.

[14] Wang J, Khoury DS, Yue Y, Torre-Amione G, Nagueh SF (2008). Preserved left ventricular twist and circumferential deformation, but depressed longitudinal and radial deformation in patients with diastolic heart failure. *European Heart Journal*.

[15] Liu Y-W, Tsai W-C, Su C-T, Lin C-C, Chen J-H (2009). Evidence of left ventricular systolic dysfunction detected by automated function imaging in patients with heart failure and preserved left ventricular ejection fraction. *Journal of Cardiac Failure*.

[16] Liu Y-W, Su C-T, Huang Y-Y (2011). Left ventricular systolic strain in chronic kidney disease and hemodialysis patients. *American Journal of Nephrology*.

[17] Stanton T, Leano R, Marwick TH (2009). Prediction of all-cause mortality from global longitudinal speckle strain: comparison with ejection fraction and wall motion scoring. *Circulation: Cardiovascular Imaging*.

[18] Liu YW, Su CT, Sung JM (2013). Association of left ventricular longitudinal strain with mortality among stable hemodialysis patients with preserved left ventricular ejection fraction. *Clinical Journal of the American Society of Nephrology*.

[19] Wang AY-M, Lam CW-K, Wang M (2007). Prognostic value of cardiac troponin T is independent of inflammation, residual renal function, and cardiac hypertrophy and dysfunction in peritoneal dialysis patients. *Clinical Chemistry*.

[20] Mallamaci F, Zoccali C, Parlongo S (2002). Troponin is related to left ventricular mass and predicts all-cause and cardiovascular mortality in hemodialysis patients. *American Journal of Kidney Diseases*.

[21] Iliou MC, Fumeron C, Benoit MO (2001). Factors associated with increased serum levels of cardiac troponins T and I in chronic haemodialysis patients: chronic Haemodialysis and new Cardiac Markers Evaluation (CHANCE) study. *Nephrology Dialysis Transplantation*.

[22] Lang RM, Bierig M, Devereux RB (2005). Recommendations for chamber quantification: a report from the American Society of Echocardiography’s guidelines and standards committee and the Chamber Quantification Writing Group, developed in conjunction with the European Association of Echocardiography, a branch of the European Society of Cardiology. *Journal of the American Society of Echocardiography*.

[23] McMurray JJ, Adamopoulos S, Anker SD (2012). ESC Guidelines for the diagnosis and treatment of acute and chronic heart failure 2012: the Task Force for the Diagnosis and Treatment of Acute and Chronic Heart Failure 2012 of the European Society of Cardiology. Developed in collaboration with the Heart Failure Association (HFA) of the ESC. *European Heart Journal*.

[24] Nagueh SF, Appleton CP, Gillebert TC (2009). Commendations for the evaluation of left ventricular diastolic function by echocardiography. *Journal of the American Society of Echocardiography*.

[25] Ando Y, Yanagiba S, Asano Y (1995). The inferior vena cava diameter as a marker of dry weight in chronic hemodialyzed patients. *Artificial Organs*.

[26] National Kidney Foundation (2006). KDOQI clinical practice guidelines and clinical practice recommendations for 2006 updates: hemodialysis adequacy, peritoneal dialysis adequacy and vascular access. *American Journal of Kidney Diseases*.

[27] Yashiro M, Kamata T, Yamadori N, Tomita M, Muso E (2007). Evaluation of markers to estimate volume status in hemodialysis patients: atrial natriuretic peptide, inferior vena cava diameter, blood volume changes and filtration coefficients of microvasculature. *Therapeutic Apheresis and Dialysis*.

[28] Yingchoncharoen T, Agarwal S, Popović ZB, Marwick H (2013). Normal ranges of left ventricular strain: a meta-analysis. *Journal of the American Society of Echocardiography*.

[29] Sharma R, Gaze DC, Pellerin D (2006). Cardiac structural and functional abnormalities in end stage renal disease patients with elevated cardiac troponin T. *Heart*.

[30] deFilippi CR, Thorn EM, Aggarwal M (2007). Frequency and cause of cardiac troponin T elevation in chronic hemodialysis patients from study of cardiovascular magnetic resonance. *American Journal of Cardiology*.

[31] Mallamaci F, Zoccali C, Parlongo S (2002). Diagnostic value of troponin T for alterations in left ventricular mass and function in dialysis patients. *Kidney International*.

[32] Burton JO, Jefferies HJ, Selby NM, McIntyre CW (2009). Hemodialysis-induced cardiac injury: determinants and associated outcomes. *Clinical Journal of the American Society of Nephrology*.

[33] Burton JO, Jefferies HJ, Selby NM, McIntyre CW (2009). Hemodialysis-induced repetitive myocardial injury results in global and segmental reduction in systolic cardiac function. *Clinical Journal of the American Society of Nephrology*.

[34] Breidthardt T, Burton JO, Odudu A, Eldehni MT, Jefferies HJ, McIntyre CW (2012). Troponin T for the detection of dialysis-induced myocardial stunning in hemodialysis patients. *Clinical Journal of the American Society of Nephrology*.

[35] Obialo CI, Sharda S, Goyal S, Ofili EO, Oduwole A, Gray N (2004). Ability of troponin T to predict angiographic coronary artery disease in patients with chronic kidney disease. *American Journal of Cardiology*.

[36] Hayashi T, Obi Y, Kimura T (2008). Cardiac troponin T predicts occult coronary artery stenosis in patients with chronic kidney disease at the start of renal replacement therapy. *Nephrology Dialysis Transplantation*.

[37] Liu Y-W, Su C-T, Wang SPH (2011). Application of speckle-tracking echocardiography in detecting coronary artery disease in patients with maintenance hemodialysis. *Blood Purification*.

[38] Ooi DS, Isotalo PA, Veinot JP (2000). Correlation of antemortem serum creatine kinase, creatine kinase-MB, troponin I, and troponin T with cardiac pathology. *Clinical Chemistry*.

[39] Ooi DS, Zimmerman D, Graham J, Wells GA (2001). Cardiac troponin T predicts long-term outcomes in hemodialysis patients. *Clinical Chemistry*.

[40] Jeon D-S, Lee M-Y, Kim C-J (2004). Clinical findings in patients with cardiac troponin T elevation and end-stage renal disease without acute coronary syndrome. *American Journal of Cardiology*.

[41] Ooi DS, House AA (1998). Cardiac troponin T in hemodialyzed patients. *Clinical Chemistry*.

[42] Dierkes J, Domröse U, Westphal S (2000). Cardiac troponin T predicts mortality in patients end-stage renal disease. *Circulation*.

[43] Deegan PB, Lafferty ME, Blumsohn A, Henderson IS, McGregor E (2001). Prognostic value of troponin T in hemodialysis patients is independent of comorbidity. *Kidney International*.

[44] Apple FS, Murakami MM, Pearce LA, Herzog CA (2002). Predictive value of cardiac troponin I and T for subsequent death in end-stage renal disease. *Circulation*.

[45] Satyan S, Light RP, Agarwal R (2007). Relationships of N-terminal pro-Bnatriuretic peptide and cardiac troponin T to left ventricular mass and function and mortality in asymptomatic hemodialysis patients. *American Journal of Kidney Diseases*.

[46] Conway B, McLaughlin M, Sharpe P, Harty J (2005). Use of cardiac troponin T in diagnosis and prognosis of cardiac events in patients on chronic haemodialysis. *Nephrology Dialysis Transplantation*.

[47] Khan NA, Hemmelgarn BR, Tonelli M, Thompson CR, Levin A (2005). Prognostic value of troponin T and I among asymptomatic patients with end-stage renal disease: a meta-analysis. *Circulation*.

[48] Löwbeer C, Gustafsson SA, Seeberger A, Bouvier F, Hulting J (2004). Serum cardiac troponin T in patients hospitalized with heart failure is associated with left ventricular hypertrophy and systolic dysfunction. *Scandinavian Journal of Clinical and Laboratory Investigation*.

[49] Geyer H, Caracciolo G, Abe H (2010). Assessment of myocardial mechanics using speckle tracking echocardiography: fundamentals and clinical applications. *Journal of the American Society of Echocardiography*.

[50] Brown J, Jenkins C, Marwick TH (2009). Use of myocardial strain to assess global left ventricular function: a comparison with cardiac magnetic resonance and 3-dimensional echocardiography. *American Heart Journal*.

[51] Antoni ML, Mollema SA, Delgado V (2010). Prognostic importance of strain and strain rate after acute myocardial infarction. *European Heart Journal*.

[52] Mor-Avi V, Lang RM, Badano LP (2011). Current and evolving echocardiographic techniques for the quantitative evaluation of cardiac mechanics: ASE/EAE consensus statement on methodology and indications: endorsed by the Japanese Society of Echocardiography. *Journal of the American Society of Echocardiography*.

[53] Feigenbaum H, Mastouri R, Sawada S (2012). A practical approach to using strain echocardiography to evaluate the left ventricle. *Circulation Journal*.

[54] Hudson MP, O’Connor CM, Gattis WA (2004). Implications of elevated cardiac troponin t in ambulatory patients with heart failure: a prospective analysis. *American Heart Journal*.

[55] Perna ER, Macín SM, Canella JPC (2005). Minor myocardial damage detected by troponin T is a powerful predictor of long-term prognosis in patients with acute decompensated heart failure. *International Journal of Cardiology*.

[56] Healey JS, Davies RF, Smith SJ, Davies RA, Ooi DS (2003). Prognostic use of cardiac troponin T and troponin I in patients with heart failure. *Canadian Journal of Cardiology*.

[57] Mignot A, Donal E, Zaroui A (2010). Global longitudinal strain as a major predictor of cardiac events in patients with depressed left ventricular function: a multicenter study. *Journal of the American Society of Echocardiography*.

[58] Nahum J, Bensaid A, Dussault C (2010). Impact of longitudinal myocardial deformation on the prognosis of chronic heart failure patients. *Circulation: Cardiovascular Imaging*.

[59] Iacoviello M, Puzzovivo A, Guida P (2013). Independent role of left ventricular global longitudinal strain in predicting prognosis of chronic heart failure patients. *Echocardiography*.

