# Supplementary material for: Independent Value of Cardiac Troponin T and Left Ventricular Global Longitudinal Strain in Predicting All-Cause Mortality among Stable Hemodialysis Patients with Preserved Left Ventricular Ejection Fraction
Source: Biomed Res Int. 2014 May 7;2014:217290. doi: 10.1155/2014/217290 (PMC4033336; doi:10.1155/2014/217290)
Supplement: Supplementary file 1 — Supplemental Table S1: Pearson correlations among Echocardiographic parameters. Supplemental Table S2: Univariate analysis of factors related to all-cause mortality. [file 217290.f1.pdf]

Supplemental Table S1 Pearson correlation between Echocardiographic parameters

		LVMi	LVEF	GLS	LSRs	CS	CSRs
LVMi	*r*	1	-0.33	0.37	0.32	0.31	0.21
	p		0.005*	0.001*	0.007*	0.01*	0.09
LVEF	*r*	-0.33	1	-0.47	-0.43	-0.50	-0.36
	p	0.005*		<0.001*	<0.001*	<0.001*	0.002*
GLS	*r*	0.37	-0.47	1	0.58	0.60	0.22
(%)	*p*	0.001*	<0.001*		<0.001*	<0.001*	0.06
LSRs	*r*	0.32	-0.43	0.58	1	0.41	0.49
(sec^{-1})	*p*	0.007*	<0.001*	<0.001*		<0.001*	<0.001*
CS (%)	*r*	0.31	-0.50	0.60	0.41	1	0.52
	p	0.01*	<0.001*	<0.001*	<0.001*		<0.001*
CSRs	*r*	0.21	-0.36	0.22	0.49	0.52	1
(sec^{-1})	*p*	0.09	0.002*	0.06	<0.001*	<0.001*	

*$p < 0.05$

Abbreviations: CS, average circumferential strain; CSRs, circumferential systolic strain rate; LVEF, left ventricular ejection fraction;; GLS, global left ventricular peak systolic longitudinal strain; IVCe, end-expiratory inferior vena cava diameter; LSRs, longitudinal systolic strain rate; LV, left ventricular; LVMi, left ventricular mass index; SRs, systolic strain rate.

Supplemental Table S2. Univariate analysis of factors related to all-cause mortality

Variables	HR (95% CI)	*p* value
Age (years)	1.03 (0.99-1.06)	0.17
Gender	1.81 (0.82-4.04)	0.16
Hemodialysis duration (years)	0.95 (0.86-1.06)	0.37
IDWG (%)	0.97 (0.78-1.22)	0.81
Kt/V	0.25 (0.04-1.79)	0.17
Heart rate (beats/min)	0.99 (0.95-1.02)	0.50
Hypertension	2.66 (1.05-6.70)	0.04*
Prevalent CAD	2.60 (1.17-5.82)	0.02*
Diabetes mellitus	2.65 (1.10-6.39)	0.03*
Dyslipidemia	1.10 (0.46-2.66)	0.83
LVH	0.36 (0.10-1.24)	0.18
Ca (mg/dL)	1.03 (0.61-1.76)	0.91
P (mg/dL)	0.94 (0.68-1.30)	0.70
hsCRP (mg/dL)	1.15 (0.96-1.38)	0.18
Albumin (g/dL)	0.14 (0.04-0.43)	0.001*
cTnTx100	1.19 (1.11-1.27)	<0.001*
IVCe (cm)	4.48 (1.21-19.92)	0.25
LVMi (gm/m^2)	1.00 (0.99-1.01)	0.23
LVEF (%)	0.94 (0.88-1.01)	0.16
E/A	0.89 (0.27-2.88)	0.84
s′ (cm/s)	0.02 (0.01-6.78)	0.72
e′(cm/s)	0.10 (0.01-5.81)	0.89

Average E/ e′	1.01 (0.96-1.07)	0.59
LAVi (ml/m^2)	1.02 (0.96-1.09)	0.56
Less negative CS (CS≥-23.3%)	2.86 (0.65-12.70)	0.18
Less negative GLS (GLS ≥-15%)	1.19 (1.06-1.33)	0.004*
Longitudinal SRs	1.96 (0.28-13.90)	0.50
Circumferential SRs	1.48 (0.56-3.92)	0.43

*$p < 0.05$

Abbreviations: CAD, coronary artery disease; CI, confidence interval; CS, average circumferential strain; cTnT, cardiac troponin T; E/ e′, early trans-mitral velocity to tissue Doppler mitral annular early diastolic velocity ratio; GLS, global left ventricular peak systolic longitudinal strain; HR, hazard ratio; hsCRP, high-sensitivity C-reactive protein; IDWG, inter-dialytic weight gain; IVCe, end-expiratory inferior vena cava diameter; Kt/V, an indicator of dialysis adequacy (K, urea clearance; t, dialysis time; V, urea distribution volume); LAVi, left atrial volume index; LVEF, left ventricular ejection fraction; LVH, left ventricular hypertrophy; LVMi, left ventricular mass index; SRs, systolic strain rate; s ′, left ventricular systolic myocardial velocity.

Supplemental Table S3. Ninety-five percent confidence intervals of Cox regression models in Table 4

	95% CI for HR							
	**Basic model**	**Basic model +** cTnTx100	**Basic model +** GLS≥-15%	**Full Model**	**Full Model + Interaction term**	**Reduced Model 1**	**Reduced Model 2 (final model)**	**final Model + Interaction term**
Albumin	0.06-0.58	0.08-1.24	0.05-0.53	0.06-0.98	0.07-1.19	0.05-0.94	0.06-0.99	0.07-1.26
CAD	1.07-6.40	1.13-6.03	0.76-5.09	0.82-5.48	0.84-5.90	0.85-5.76	--	--
DM	1.11-8.80	0.46-7.97	1,06-8.31	0.56-5.33	0.47-4.74	--	--	--
HTN	1.24-9.28	1.00-8.66	1.17-8.19	1.09-8.75	0.87-8.03	1.06-8.49	0.96-7.63	0.80-6.72
cTnTx100	-	1.06-1.27	-	1.03-1.24	1.04-1.36	1.06-1.27	1.05-1.24	1.09-1.36
GLS≥-15%		--	1.41-9.04	1.14-8.43	0.88-18.24	1.23-9.20	1.01-7.77	1.51-27.44
(cTnTx100):(GLS≥-15%)[#]					0.78-1.16	--		0.78-1.14

#(cTnTx100):(GLS≥-15%), interaction between cTnTx100 and GLS≥-15%.

Abbreviations: CAD, coronary arterial disease; cTnT, cardiac troponin T, DM, diabetes; GLS, global left ventricular peak systolic longitudinal strain (less negative GLS was defined by GLS≥-15%).
